# Fabrication of tri-layered electrospun polycaprolactone mats with improved sustained drug release profile

**DOI:** 10.1038/s41598-020-74885-1

**Published:** 2020-10-23

**Authors:** S. Manjunath Kamath, K. Sridhar, D. Jaison, V. Gopinath, B. K. Mohamed Ibrahim, Nilkantha Gupta, A. Sundaram, P. Sivaperumal, S. Padmapriya, S. Shantanu Patil

**Affiliations:** 1 Department of Translational Medicine and Research, SRM Medical College, SRMIST, Kattankulathur, Tamil Nadu 603203 India; 2 Department of Medical Microbiology, Faculty of Medicine, University of Malaya, 50603 Kuala Lumpur, Malaysia; 3 Department of Pathology, SRM Medical College, SRMIST, Kattankulathur, Tamil Nadu 603203 India; 4 Nanotechnology Research Center (NRC), SRMIST, Kattankulathur, Tamil Nadu 603203 India; 5 Institute of Craniofacial, Aesthetic & Plastic Surgery (ICAPS), SRM Institute for Medical Sciences (SIMS), Chennai, Tamil Nadu 600026 India; 6 Department of Pharmacology, Saveetha Dental College (SDC), Saveetha Institute of Medical and Technical Sciences, Chennai, Tamil Nadu India; 7 Electrochemical Systems Laboratory, SRM Research Institute, SRMIST, Kattankulathur, Tamil Nadu 603203 India

**Keywords:** Biotechnology, Cell biology, Materials science

## Abstract

Modulation of initial burst and long term release from electrospun fibrous mats can be achieved by sandwiching the drug loaded mats between hydrophobic layers of fibrous polycaprolactone (PCL). Ibuprofen (IBU) loaded PCL fibrous mats (12% PCL-IBU) were sandwiched between fibrous polycaprolactone layers during the process of electrospinning, by varying the polymer concentrations (10% (w/v), 12% (w/v)) and volume of coat (1 ml, 2 ml) in flanking layers. Consequently, 12% PCL-IBU (without sandwich layer) showed burst release of 66.43% on day 1 and cumulative release (%) of 86.08% at the end of 62 days. Whereas, sandwich groups, especially 12% PCLSW-1 & 2 (sandwich layers—1 ml and 2 ml of 12% PCL) showed controlled initial burst and cumulative (%) release compared to 12% PCL-IBU. Moreover, crystallinity (%) and hydrophobicity of the sandwich models imparted control on ibuprofen release from fibrous mats. Further, assay for cytotoxicity and scanning electron microscopic images of cell seeded mats after 5 days showed the mats were not cytotoxic. Nuclear Magnetic Resonance spectroscopic analysis revealed weak interaction between ibuprofen and PCL in nanofibers which favors the release of ibuprofen. These data imply that concentration and volume of coat in flanking layer imparts tighter control on initial burst and long term release of ibuprofen.

## Introduction

Electrospinning has attracted extensive attention in the recent years, due to its facile nature and ability to rapidly synthesize fibrous mats for applications such as tissue engineering, wound dressing, filtration devices and drug delivery^1–3^. Electrospun mats provide a platform for the direct incorporation and sustained delivery of growth factors, drugs, bioactive molecules etc., for a prolonged period. In addition, properties such as high surface area to volume ratio, high drug loading and encapsulation efficiency, ability to modulate release and its cost effectiveness, make electrospun fibrous mats advantageous in drug delivery applications^1,3^. Moreover, several studies on fibrous mats eluting various growth factors, drugs, bioactive molecules as nerve conduits in spinal cord injury in vivo with promising outcomes have been reported^4^. In electrospinning, amongst various polymers used for biomedical applications, Polycaprolactone (PCL) is the highly preferred and extensively evaluated polymer in the long term drug delivery applications due to its biodegradability, biocompatibility, high mechanical strength and prolonged biodegradation time^5^.

Ibuprofen (IBU), a non-steroidal anti-inflammatory drug (NSAID) is widely used for various clinical indications to reduce inflammation and pain. In addition, there are ample reports suggesting the neuroprotective and neuroregenerative role of ibuprofen^6–9^. Hence delivery of ibuprofen in a controlled fashion would be beneficial for multiple clinical indications. However, due to the high lipophylicity of Ibuprofen, its solubility in aqueous medium is sparing. To overcome this limitation, studies on esterification of carboxylic group in ibuprofen with amino acids has been reported^10^. However, Nano-delivery systems have proven to be promising tools for delivery of such drugs wherein, amorphization during its encapsulation, improves dissolution of these drugs. Recent studies have reported that the functionalization of IBU in layered double hydroxide (LDH) nanoparticles and entrapping the IBU-LDH nanoparticles into PCL nanofibrous mats improves the solubility of drug and controls the initial burst release^11^. Moreover, poorly water soluble drugs like carvedilol and IBU loaded into PCL nanofibrous mats for oromucosal delivery suggested an increase in dissolution^12^. To overcome the problem of dissolution and release of poor drug releasing ability, RGD based cationic polymers have also been reported in the literature^13^. Hence, polymeric encapsulations like scaffolds, hydrogels^14^, nano-carriers and micro-carriers are beneficial to overcome the bottleneck of dissolution and controlled release of hydrophobic drugs^15–17^. Encapsulation of hydrophobic drugs into nanofibrous PCL, owing to its potential as drug delivery vehicles, enhances dissolution, whereas its hydrophobic property efficiently modulates rate of water diffusion and leaching out of drugs from these fibers, for sustained long term release^18^.

Various studies have reported the merits of coaxial/tri-axial fabrication of nanofibrous for sustained release of drugs^3,19–21^. Although such methods yield efficient nanofiberous systems for controlled drug release, the complexity of fabrication process is a drawback. Hence there is a need to develop straightforward method with similar or better drug release profiles. Sandwich nanofibrous models are very simple to fabricate which can serve to release drugs in a controlled fashion. In this context, researchers have developed temperature controlled release of neuronal growth factor from microparticles sandwiched between PCL nanofibrous mats to promote neurite out growth^22^. In addition, previous studies have reported a sandwich model of poly(lactic-co-glycolic acid) and collagen scaffold (PLGA/collagen) releasing vancomycin, gentamicin and lidocaine^23^ and electrospun membranes composed of polycaprolactone (PCL), shellac (PCL/shellac) releasing salicylic acid^24^ in a controlled manner. Very recently, a study reported fabrication of tri-layered electrospun with sustained release of acyclovir^25^. Importantly, Immich et al. reported high initial burst release of IBU and considerably short term release kinetics in the sandwich model of polylactic acid-IBU (PLA/IBU)^26^,wherein IBU powder was directly layered in between PLA nanofibrous membrane. Hence, it would be beneficial to encapsulate IBU into a fibrous PCL layer flanked by PCL fibrous layers rather than adding IBU directly for long term sustained release. In addition, several other studies have reported on successful loading of IBU in single layered electrospun mats and particulate nano delivery systems having high burst release of IBU^27–32^. To the best of knowledge, there are no much reports on sandwich model of IBU encapsulated in PCL fibrous mat carrying flanked layers of PCL. Thus the major objective of the present work are as follows (i) electrospinning of fibers eluting IBU flanked between PCL fibers for sustained and prolonged release of IBU during the process of electrospinning. (ii) Physicochemical characterization to study chemical changes during fabrication. (iii) Cumulative(%) release and drug release kinetics evaluation. (iv) Cytotoxicity analysis in vitro. The composition and labels for each group in the study are given below in Table 1.

**Table 1:** Composition of sandwich and non sandwich mats.

Groups	Bottom layer	Middle layer	Top layer
10% PCLSW- 1	10%PCL, 1 ml coat	12% PCL-IBU Loaded, 2 ml coat	10% PCL, 1 ml coat
10% PCLSW- 2	10%PCL, 2 ml coat	12% PCL- IBU Loaded, 2 ml coat	10% PCL, 2 ml coat
12% PCLSW- 1	12%PCL, 1 ml coat	12% PCL-IBU Loaded, 2 ml coat	12% PCL, 1 ml coat
12% PCLSW- 2	12%PCL, 2 ml coat	12% PCL-IBU Loaded, 2 ml coat	12% PCL, 2 ml coat
12% PCL- IBU	Single layer, 2 ml coat without flanking layers		

## Results

### Morphological analysis

**Figure 1:**
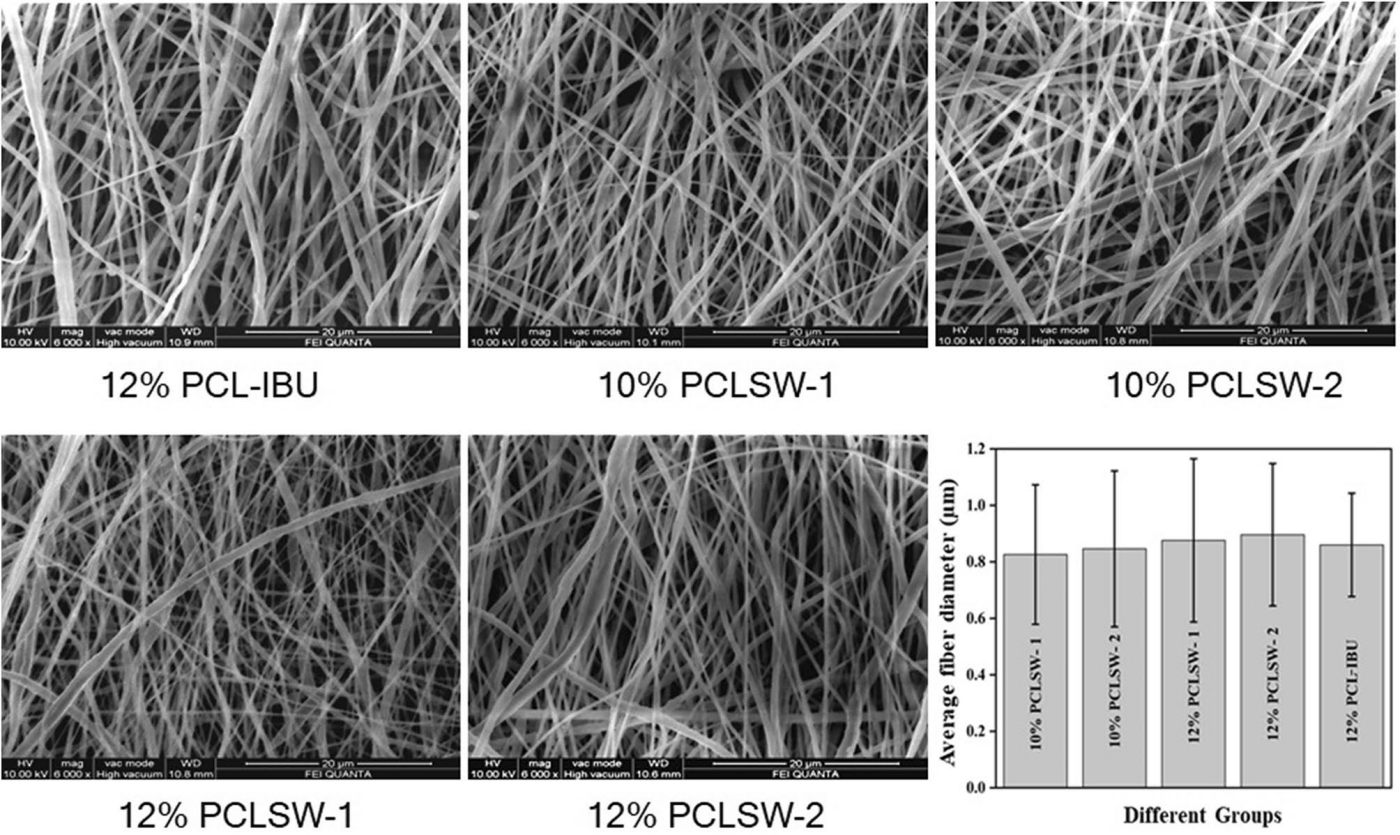
FESEM images. Morphology of electrospun fibrous mats of different groups and average fiber diameter with standard deviation. *PCL* polycaprolactone, *PCLSW* polycaprolactone sandwich mat of different groups, *IBU* ibuprofen).

FESEM images are shown in Fig. 1. Image J software was used to measure the fiber diameters at 5 different areas on the image. The average fiber diameters and their standard deviations were found to be 826 ± 247 nm for 10% PCLSW-1, 846 ± 276 nm for 10% PCLSW-2, 876 ± 289 nm for 12% PCLSW-1, 896 ± 252 nm for 12% PCLSW-2 and 860 ± 183 nm for 12% PCL-IBU. Fiber diameter distribution (Supplementary Fig. S1) of the groups was in the range of 400 nm–1.2 µm. The increase in fiber diameters of the sandwich fibrous mats are attributed to the increasing volume of the flanking non-conductive polycaprolactone coat (4 ml to 6 ml) which causes decrease in electric field intensity thereby diminishing the whipping and stretching of polymer jet towards grounded collector^33,34^. However, 2 ml coat of 12% PCL-IBU also showed average fiber diameter in the same range as that of sandwich mats. This is consequential of adding IBU which could have interfered with the whipping and stretching of polymer jet due to its covalent nonconductive nature.

### XRD analysis

**Figure 2:**
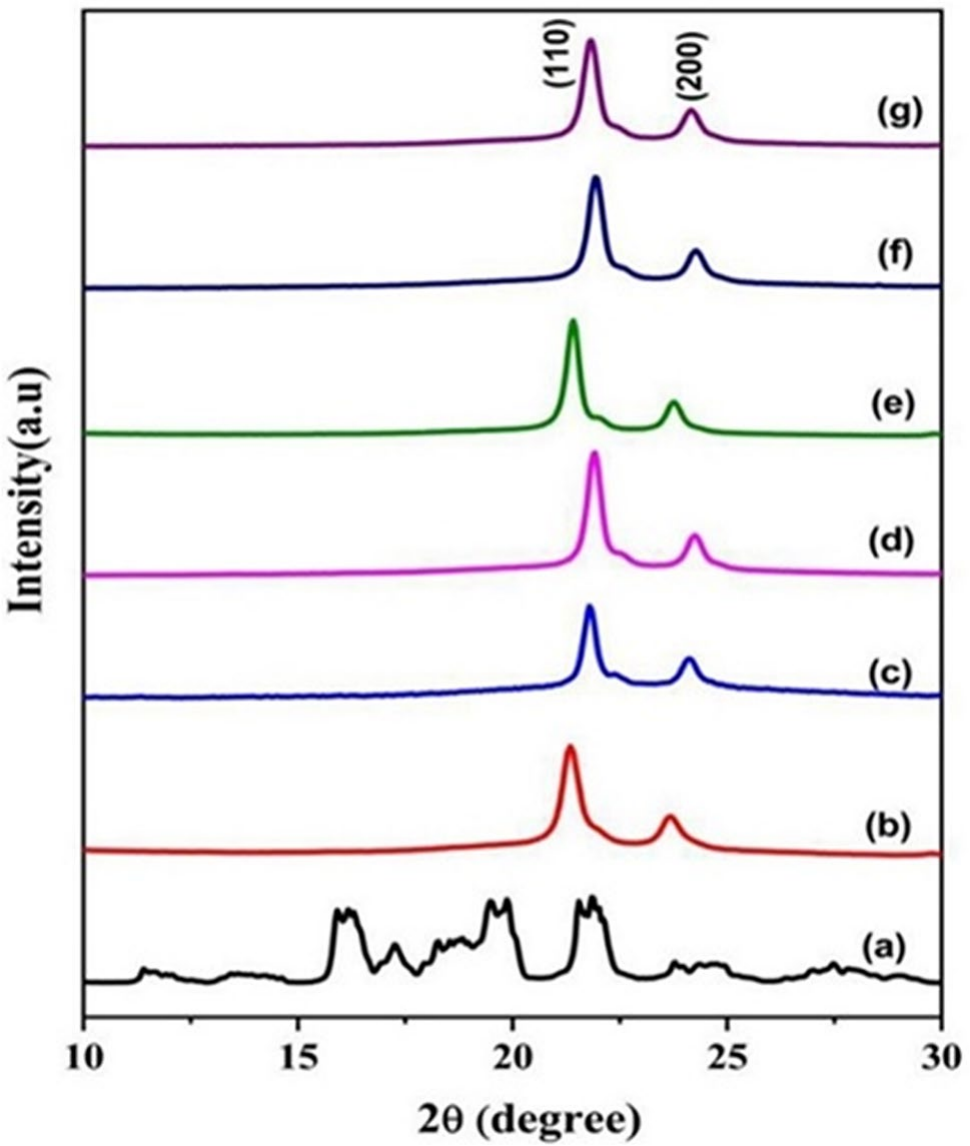
XRD spectra of fibrous mats. **(a)** IBU **(b)** 12%PCL mat (without drug) **(c)** 12% PCL-IBU **(d)** 10% PCLSW- 1 **(e)** 10% PCLSW- 2 **(f)** 12% PCLSW- 1 **(g)** 12% PCLSW- 2.

XRD confirms the drug induced changes in the crystallinity of electrospun fibrous mats as shown in Fig. 2. XRD measurement reveals two well resolved peaks observed at 2θ, of 21.9° and 24.2° indexed to main peak at (110) and (200) lattice planes respectively of an orthorhombic crystalline structure of PCL^35^. There are no additional characteristic peaks corresponding to IBU observed in the drug loaded fibrous mats due to complete dissolution and amorphization^36^ during fabrication process. Further, minor shifts in the 2θ position of both peaks were observed for all groups (Supplementary Fig. S2) indicating interaction of IBU with the lattice of PCL at the molecular level, causing disorderliness in polymeric chain arrangements. Further, fiber alignment, diameter, and concentration of the polymer may have also contributed to the peak shifts^37^. The calculated crystallinity (%) and crystallite sizes of IBU loaded mats are given in Supplementary Table S1. 12% PCLSW-1&2 were found to contain crystallite sizes smaller than 10% PCLSW-1&2 as well as 12% PCL-IBU. Despite larger crystallite size of 12% PCL-IBU, the crystallinity (%) was found to be considerably lower than sandwich groups.

### Raman and FTIR spectroscopic analysis

**Figure 3:**
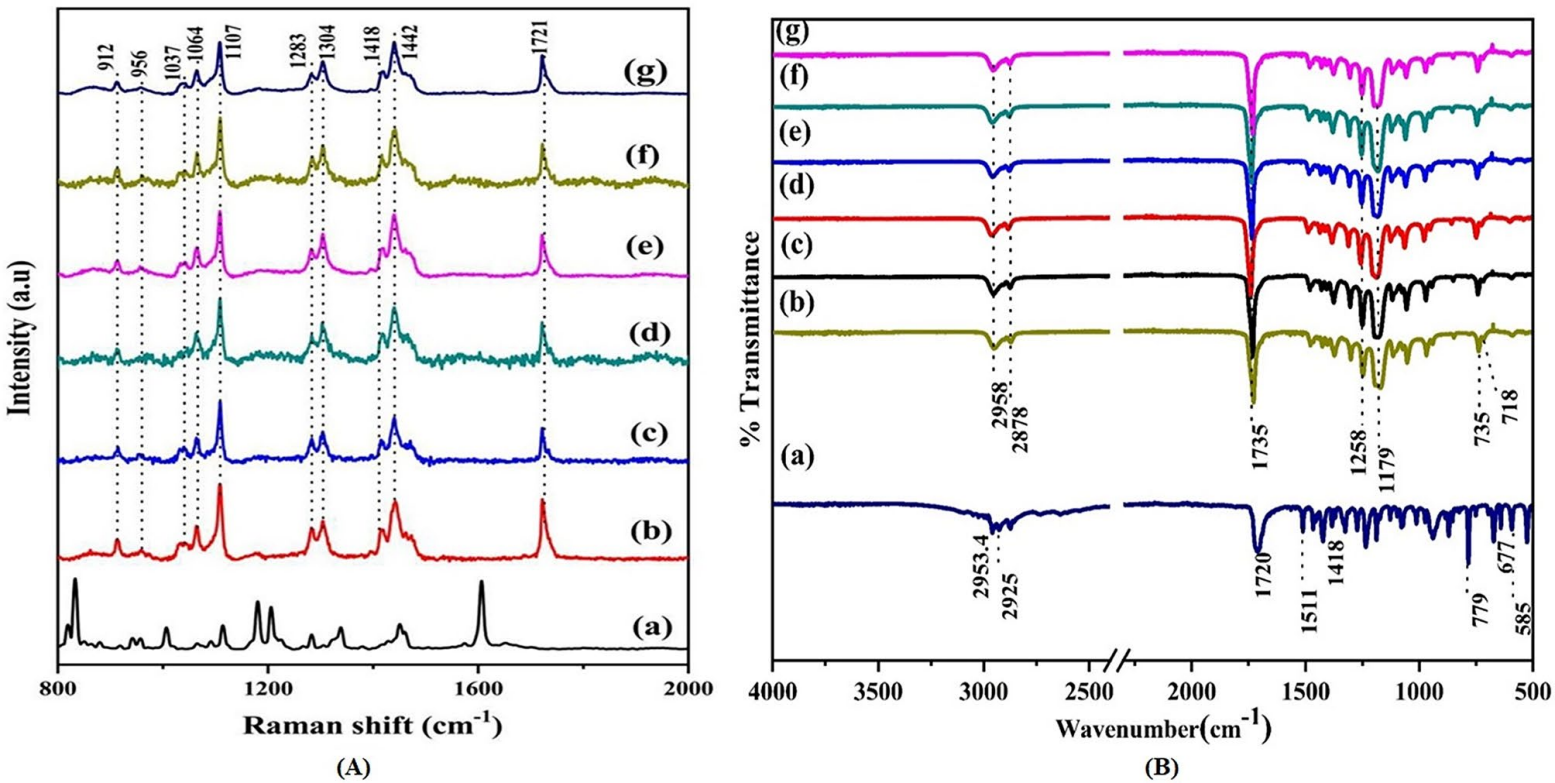
**(A)** Raman spectra for **(a)** IBU **(b)** 12% PCL mat (without drug) **(c)** 12% PCL-IBU **(d)** 10% PCLSW-1 **(e)** 10% PCLSW-2 **(f)** 12% PCLSW- 1 **(g)** 12% PCLSW-2. (Peak values in Red shows IBU peaks) and **(B)** FTIR spectra of **(**a**)** IBU **(b)** 12% PCL mat (without drug) **(c)** 12% PCL-IBU **(d)** 10% PCLSW- 1 **(e)** 10% PCLSW- 2 **(f)** 12% PCLSW- 1 **(g)** 12% PCLSW- 2.

To understand in more detail, the structural variations of the samples, Raman spectroscopic analysis were performed. The Raman spectrum exhibited typical finger-print of PCL in the region 800–2000 cm^−1^ as shown in Fig. 3A. All samples (Fig. 3A(b-g)) exhibited distinctive peaks in the C-COO skeletal stretching modes (υC–COO) at 912 cm^−1^, 956 cm^−1^ and C–C stretching modes (υ C–C ) at 1037 cm^−1^ , 1064 cm^−1^, 1107 cm^−1^. A doublet peak at 1283 cm^-1^ and 1304 cm^−1^, occurred due to twisting of CH_2_ (ωCH_2_) group derived from crystalline and amorphous PCL planes respectively^38^. The CH_2_ bending vibration specific to methylene linkages (δCH_2_) are located at 1418 and 1442 cm^−1^. A strong carbonyl stretching mode (υC=O) at around 1721 cm^−1^ was observed. The Raman spectrum showing such sharp peaks in these regions are attributed to the crystallinity of the sample. The mode positional dependence of all the samples are summarized in Supplementary Table S2^38,39^. The magnified images of strong peaks observed in the methylene bending region at 1442 cm^−1^ and the carbonyl stretch region at 1721 cm^−1^ are shown in Supplementary Fig. S3. These regions show a minor peak shift along the Raman peak position for 12% PCL-IBU compared to 12% PCL fibrous mat (without drug). For the carbonyl stretch region, the peak shift is towards lower frequency regions from 1731 cm^−1^ to 1736 cm^−1^ in 12% PCL mat (without drug) to 1730 and 1734 cm^−1^ in 12% PCL-IBU which is attributed to weak interactions between functional groups of PCL and IBU such as Van der Waals or H-bonding lowering the vibrational frequency of the C=O bond^39^.

Further, FTIR spectra for all groups are shown in Fig. 3B. The functional modes of all groups under study (10% PCLSW-1, 10% PCLSW-2, 12% PCLSW-1, 12% PCLSW-2, 12% PCL-IBU), were almost similar and consistent with 12% PCL (nanofibrous mat without drug). The bands at 2958 and 2878 cm^−1^ exhibits CH_2_ asymmetric and symmetric stretching whereas 1735, 1258, 1179, 735 and 718 cm^-1^ are due to C=O, and C–O stretching in the crystalline phase, C–O–C asymmetric stretching, C–COO crystalline phase and CH_2_ rocking respectively^40^. In this study we infer that all drug loaded mats show similar functional groups with same wavenumber as for PCL. Also, there were no additional peaks and shift in peaks of PCL noted in IBU loaded groups implying that IBU remained chemically inert to fabrication process and the interaction is very weak such as Vander Waals or H—bonding as supported by ^1^HNMR and Raman analysis.

### ^1^HNMR analysis

**Figure 4:**
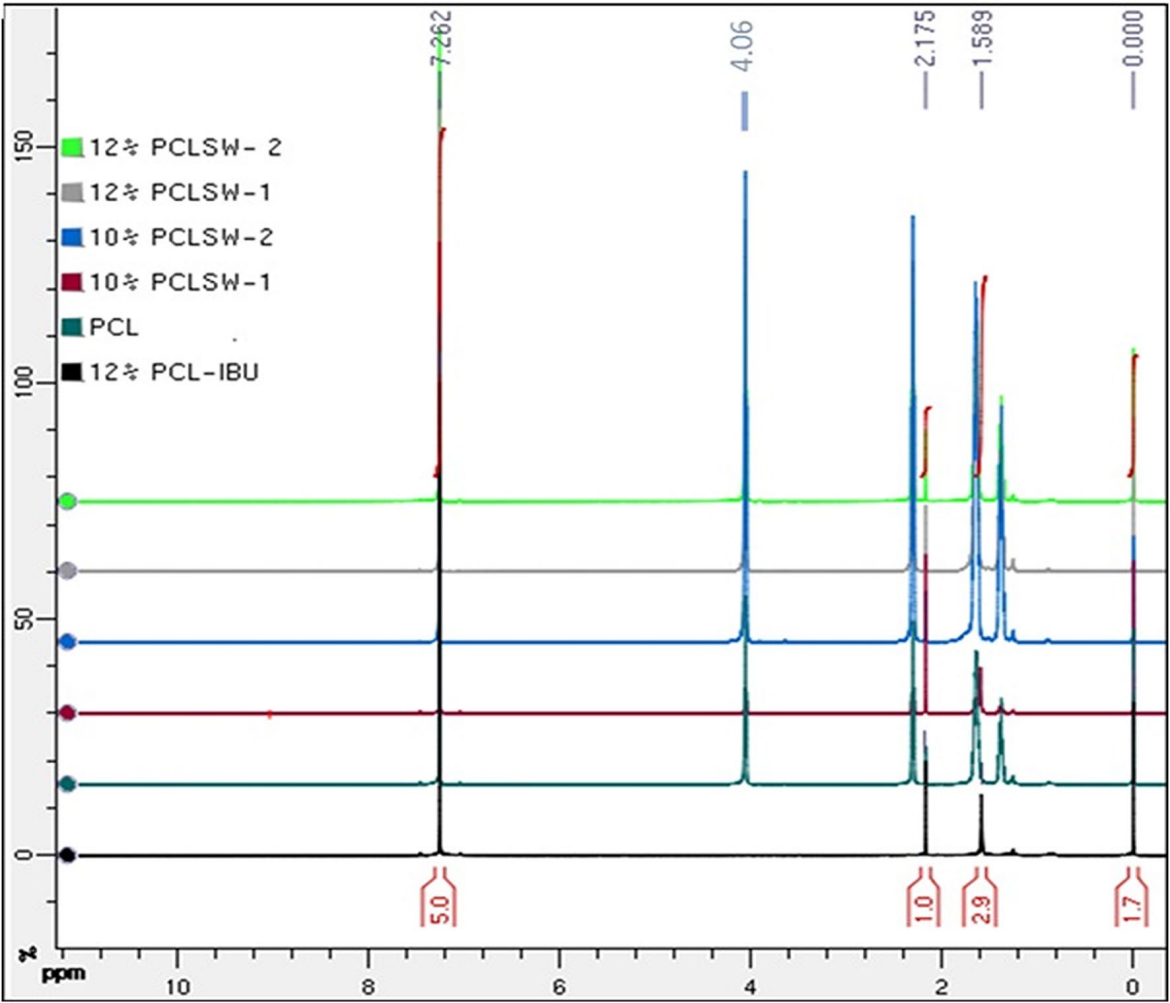
^1^HNMR Spectra of all group.

Figure 4 showed the ^1^HNMR spectra of various sandwich and non-sandwich mats in CDCl_3._^1^H residual solvent chemical shift at 7.26 ppm for CDCl_3_ and the peak at 0 ppm denotes TMS peak for all type of PCL mat with and without drug. PCL mat (without IBU) exhibited triplet peak at 4.0 and 2.3 ppm due to the protons of methylene group adjacent to the ester oxygen of PCL in addition to a sharp singlet at 2.175 ppm. Moreover, multiplet peaks were observed in the ranges of 1.8–1.6 ppm and 1.4–1.2 ppm due to the protons of methylene group adjacent to the ester oxygen of PCL. However, in sandwich groups (10% PCLSW-1, 10% PCLSW-2, 12% PCLSW-1 and 12% PCLSW-2) similar peaks were observed with a marked change in the intensity of peaks implying weak Vander Waals or H–Bonding of PCL with IBU.

**Figure 5:**
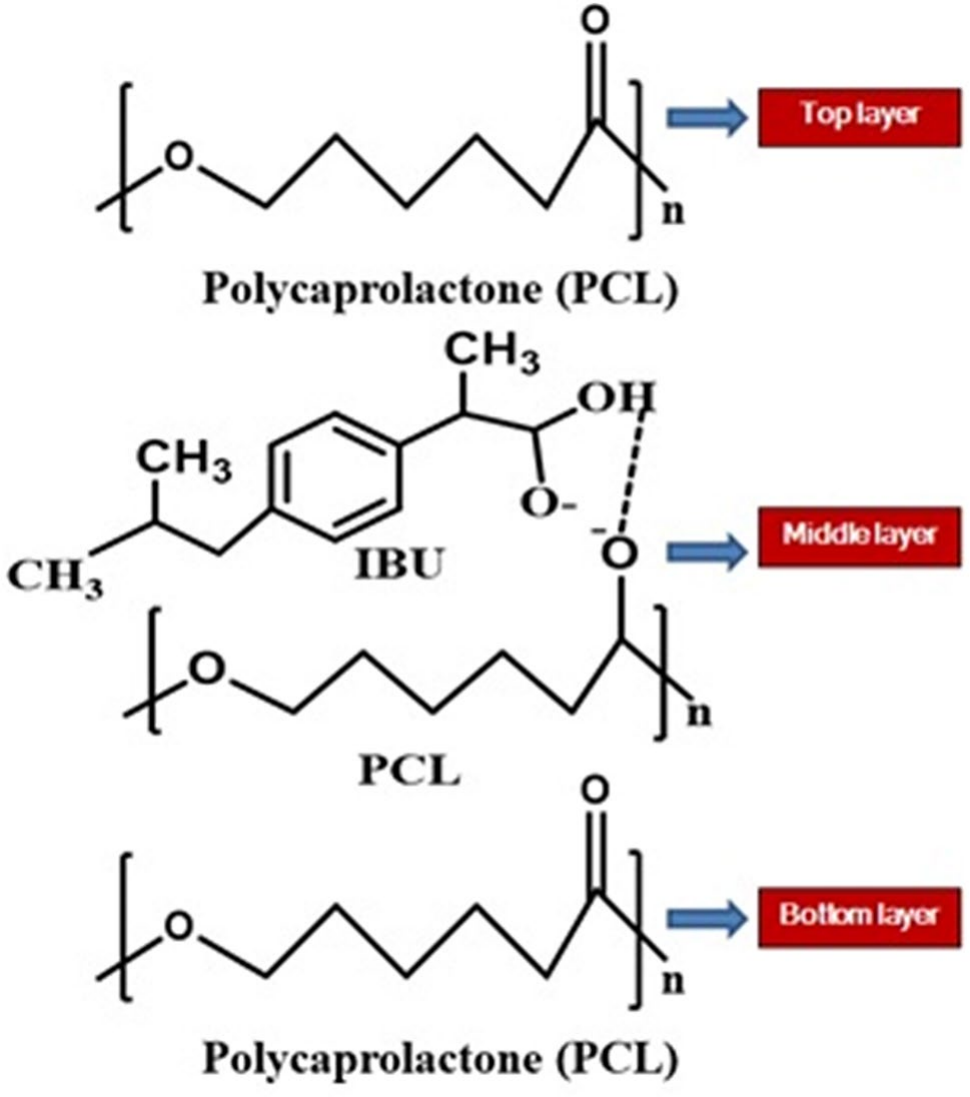
Schematic representation of IBU interaction with PCL in fibrous mats.

The Schematic representation of IBU interaction with PCL is illustrated in Fig. 5. In 12% PCL-IBU, triplet peak at 4.0 ppm and multiplet peaks at 1.8–1.6, 1.4–1.2 was not found. However, singlet peak appears at 2.175 and 1.589 ppm due to the hydrogen from the IBU bonded with PCL. In general, carboxylic acid group from IBU around 11–12 ppm was absent for all IBU loaded groups (10% PCLSW-1, 10% PCLSW-2, 12% PCLSW-1 and 12% PCLSW-2) indicating the drug is well packed into nanofibers, which could modulate for controlled initial burst and long term release. Further, it was also observed in Raman and FTIR analysis there is only minor peak shifts between PCL with and without drug. All these studies emphasize on the fact that the interaction between PCL and IBU is considerably weak, therefore advantageous for IBU release from the fibrous matrix.

### Contact angle measurements

**Figure 6:**
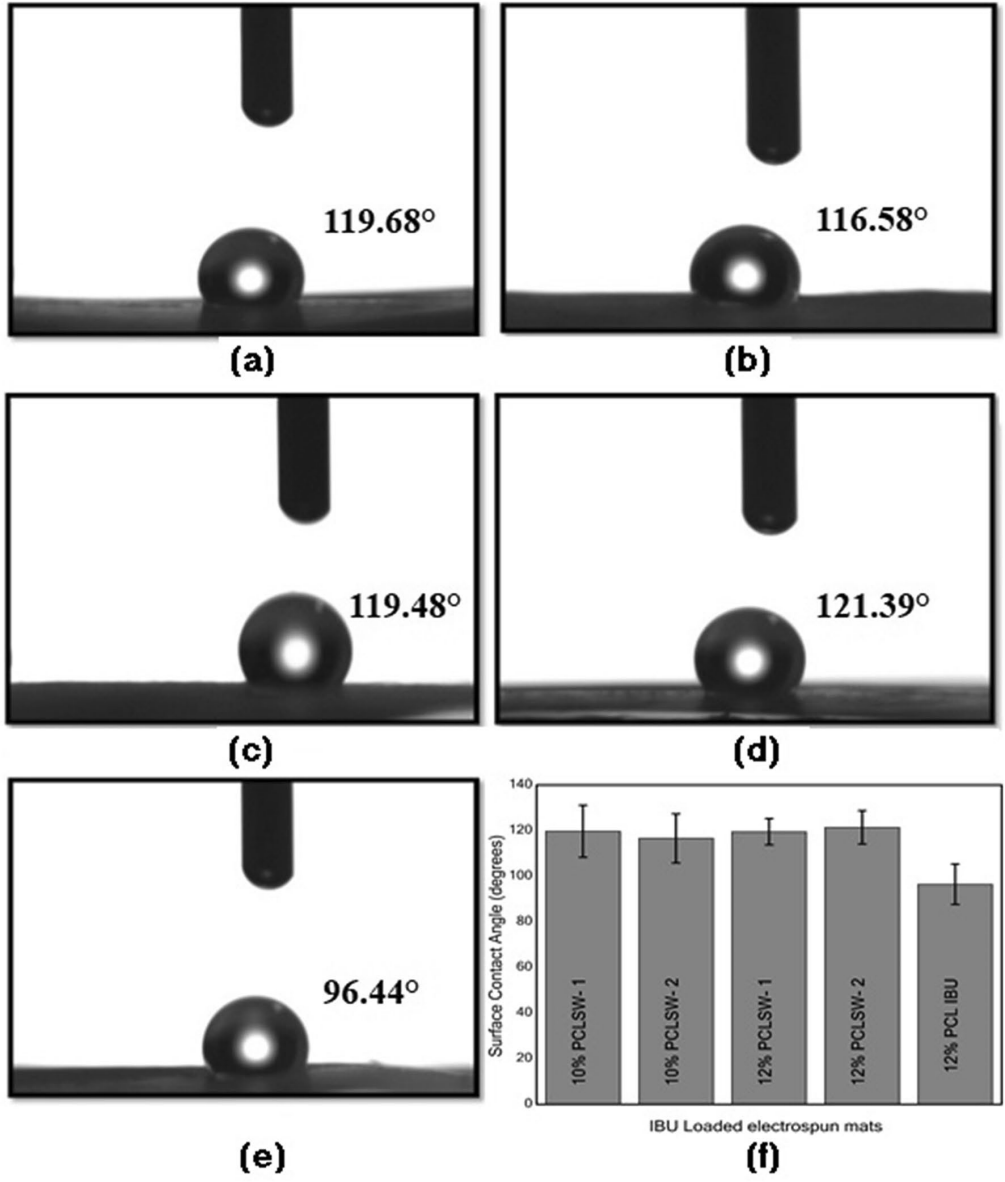
Contact angle measurements of **(a)** 10% PCLSW-1 **(b)** 10% PCLSW-2 **(c)** 12% PCLSW- 1 **(d)** 12% PCLSW-2 **(e)** 12% PCL-IBU and **(f)** average contact angles for all groups with standard deviation.

The angles of contact for all groups measured with distilled water drop are shown in Fig. 6. The data obtained suggests that 12% PCL-IBU showed least hydrophobicity (96.4°) amongst the group. However, 12% PCLSW-1, 12% PCLSW-2, 10% PCLSW-1, and 10% PCLSW-2 showed almost similar hydrophobicity in the range of 116° to 121° but were significantly higher compared to 12% PCL-IBU. The one way ANOVA for statistical significance showed that P values for groups 12% PCLSW-1, 12% PCLSW-2, 10% PCLSW-1, 10% PCLSW-2 were 0.004, 0.016, 0.005, 0.0025 respectively with 12% PCL-IBU. Previous studies suggested that the concentration of the polymer^41^, mat porosity^41^, surface roughness^42^, crystallinity and alignment of fibers^43^ influence the hydrophobic properties of electrospun fibrous surface. Hence, low hydrophobicity of 12% PCL-IBU is ascribed to its considerably lesser polymer coating compared to all other mats which consequently could have reduced fiber density and increased porosity of the mat. In addition, the crystallinity (%) of 12% PCL-IBU has decreased substantially in comparison to other sandwich groups which lead to the decrease in hydrophobicity^43^.

### Cumulative drug release analysis

**Figure 7:**
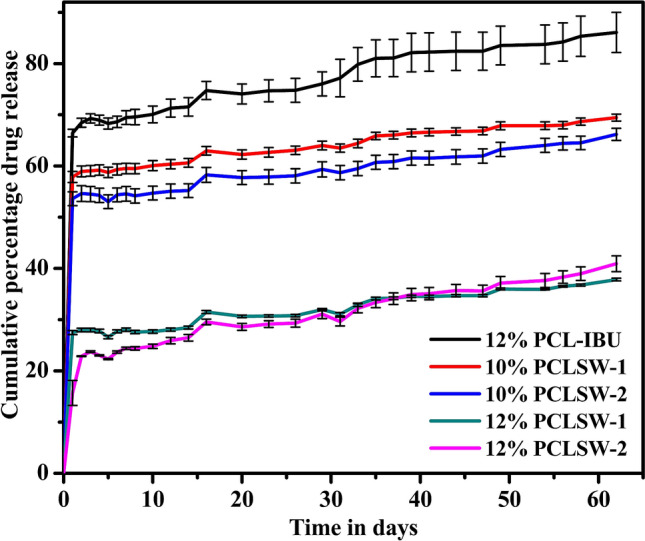
Cumulative (%) IBU release for 62 days. Cumulative (%) release comparison for all groups. (*PCL* polycaprolactone, *PCLSW* Polycaprolactone sandwich mat of different groups, *IBU* ibuprofen).

In this investigation, the cumulative release (%) of IBU from each group at a given time has been studied. From Fig. 7, it is evident that the cumulative % of IBU released were 86.08%, 69.45%, 66.18%, 37.83% and 40.93% for 12% PCL-IBU, 10%PCLSW-1, 10%PCLSW-2, 12%PCLSW-1 and 12%PCLSW-2 respectively. Also, the initial burst release was 66.43%, 57.87%, 53.61%, 27.48%, and 15.70% respectively for above mentioned groups. The initial loading of drug calculated from random sampling showed variation amongst groups due to innate disadvantage of loss of fibers during electrospinning process which leads to uneven coating.

### Release Kinetics modeling

The cumulative average drug release kinetics of IBU from PCL was modeled via (i) First order, (ii) Korsmeyer-Peppas (iii) Hixson-Crowell and (iv) Higuchi diffusion, shown as plots in Supplementary Figs. S4–S8. Further, Table 2 indicates the type of kinetics followed by all the group of samples studied in the present work. According to the R^2^ values obtained for various models, it was found that all groups followed Korsmeyer Peppas kinetics with “n” value ranging between 0.047 and 0.19 indicating the Fickian diffusion from the matrix. Fickian diffusion mechanisms are observed when the polymer relaxation rate is much greater than solvent diffusion rate^44^. The hydrophobic property of PCL could be the cause for retardation of solvent diffusion and wetting of the fibers, thereby minimizing the leaching of drug from the matrix. Moreover, from the Table 2, it is found that the correlation coefficient of the Higuchi diffusion kinetics increases with an increase in concentration and volume of PCL in the flanking layer. Consequently, applying kinetics to the fibrous matrix systems revealed the controlled diffusion mechanism of drug release, which is regulated by the hydrophobic PCL concentration and volume of polymer coat wrapping the middle layer carrying IBU.

**Table 2:** The R^2^ values for Linear fit of Cumulative release kinetics into different models.

Groups	First Order Kinetics	Korsmeyer Peppas		Hixson Crowell	Higuchi diffusion
	R^2^	n	R^2^	R^2^	R^2^
10% PCLSW-1	0.4491	0.0474	0.8595	0.3824	0.405
10% PCLSW-2	0.3522	0.053	0.7968	0.3379	0.4427
12% PCLSW-1	0.5781	0.0877	0.7798	0.5490	0.6175
12% PCLSW-2	0.8233	0.1899	0.9232	0.8016	0.8658
12% PCL-IBU	0.7357	0.0614	0.8183	0.6206	0.5221

### Cytotoxicity analysis

**Figure 8:**
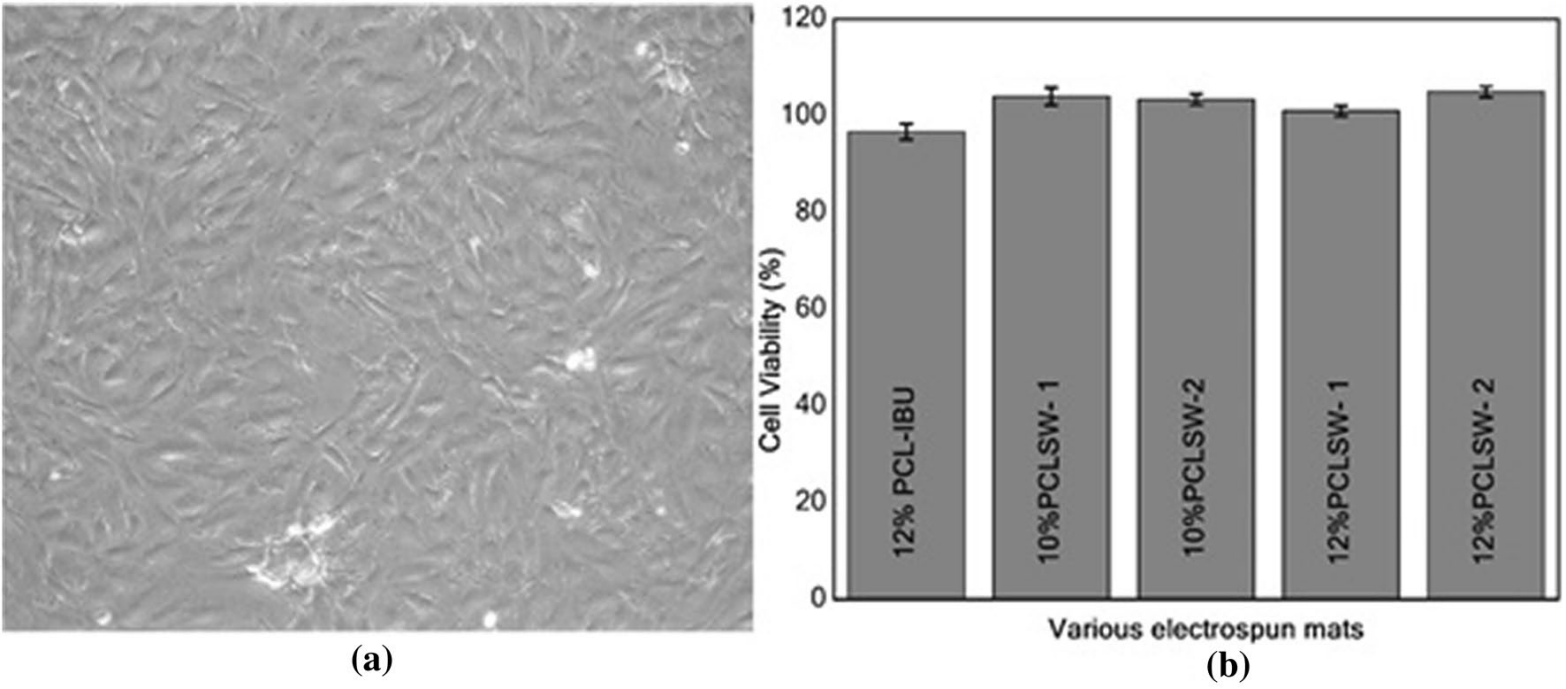
Assay for cytotoxicity **(a)** Inverted microscopic image (10 ×) of C3H10T1/2 (Murine mesenchymal stem cells) at confluence **(b)** MTT assay absorbance for different groups with standard deviation (*PCL* polycaprolactone, *PCLSW* polycaprolactone sandwich mat).

**Figure 9:**
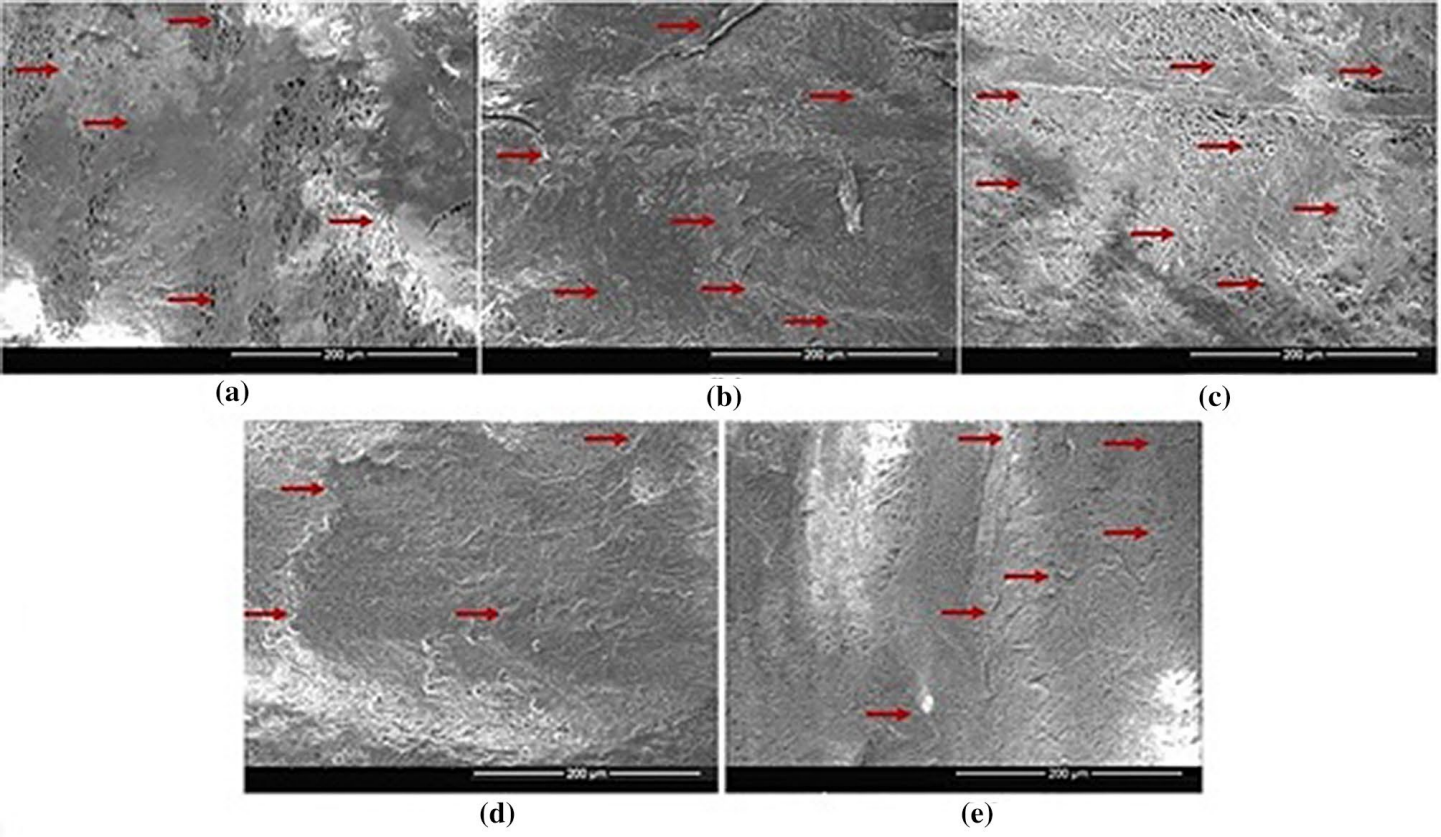
SEM images of C3H10T1/2 (Murine mesenchymal stem cells) cultured on different groups for 5 days showing growth of continuous layer of cells. **(a)**12% PCL-IBU, **(b)** 10%PCLSW-1, **(c)** 10% PCLSW-2, **(d)** 12% PCLSW-1, **(e)** 12% PCLSW-2.

Figure 8a shows the confluent layer of C3H10T1/2 cells which were used for cytotoxic and SEM analysis. The results of MTT (cell viability (%)) are shown in Fig. 8b. Analysis of variance (ANOVA) was performed on the obtained absorbance data which suggests that there was no significant (P > 0.1) difference between groups. Further, cell viability (%) was calculated which implies non-cytotoxicity of all sandwich groups with an inconsequential decrease in viability (%) in 12% PCL-IBU group. Our results are consistent with previous studies which reported non-cytotoxicity of IBU loaded carriers in vitro^45–47^*.* The non-cytotoxic nature of the IBU loaded mat was further demonstrated with SEM images of cells grown on the mats of different groups for 5 days as shown in Fig. 9. SEM images showed that cells were attached and proliferated, which are evident from the continuous monolayer of cells covering the surface of the mat. Also, PCL is FDA approved polymer and IBU is extensively studied and prescribed in clinical practice with a few known side effects, these IBU loaded mats can further be used in vivo for its efficacy.

## Discussion

In this study, it was noted that the crystallite sizes of 10% PCLSW-1, 10% PCLSW-2 and 12%PCL-IBU were larger compared to 12% PCLSW-1 and 12% PCLSW-2. This decrease in crystallite sizes of 12% PCLSW-1&2 is attributed to the higher concentration and viscosity of the polymer which increases entangling and disorderliness of polymeric chains leading to smaller lamellae formation during electrospinning. However, 12% PCL-IBU showed decreased crystallinity compared to other groups due to the interference of IBU with polymeric chains at molecular level via weak interactions such as Vander Waals or H-bonding as suggested by FTIR, Raman and ^1^HNMR studies. Similar decrease in crystalinity due to addition of drug into nanofibers is reported earlier in Cilostazol loaded PCL nanofibers^48^. Further, 10% PCLSW-1&2 showed a marginal increase in crystallinity percentage compared to 12% PCLSW-1&2 which is in agreement with the previous reports that an increase in concentration of the polymer decreases crystallinity (%)^44^. Despite smaller crystallite sizes of 12% PCLSW-1&2 mats compared to 12% PCL-IBU, their calculated crystallinity (%) showed a considerable increase, which is due to the compounded effect of flanking layers of PCL. Importantly, the crystalline peaks of IBU were not observed in any of the groups implying amorphization of sparingly water soluble drug, thereby enhancing the dissolution which is highly advantageous for drug delivery. Further, from Raman spectra of 12% PCL-IBU, it was inferred that the incorporation of IBU into PCL considerably increased stress in polymeric chain and contributed to the interference in vibrational frequency of Raman peaks at methylene and carbonyl regions of PCL. Moreover, 12% PCL-IBU did not show additional peaks compared to 12% PCL (without drug) which confirms that there was no strong bonding between IBU and the polymer as supported by ^1^HNMR and FTIR data. Similarly, FTIR and Raman did not show IBU peaks in 12%PCL-IBU due to low weight (%) compared to polymer weight^49^. Hence, sandwich fibrous mats with relatively higher crystallinity (%) along with amorphized IBU, embedded into polymeric chains without strong bonding, is advantageous for drug delivery application.

12% PCLSW-1&2 showed better sustained long term release of IBU compared to 10% PCLSW-1&2 and 12% PCL-IBU. Moreover, low crystallinity (%) and low hydrophobicity of 12% PCL-IBU enhanced rapid release of IBU leading to upsurge in the initial burst. All sandwich mats were highly hydrophobic, elucidating the modulation of initial burst release by virtue of the flanking hydrophobic coat. Hydrophobicity plays a crucial role in drug delivery for sustained and extended release of drugs, as it decreases wettability of the matrix thereby retarding the release of drug which is in agreement with previous reports^26,41^. Moreover, earlier reports also suggest the importance of crystallinity (%) of polymer in drug release modulation patterns^34,40,50^ by virtue of its highly arranged crystalites barricading the peneteration of aqueous medium. These results suggest that initial burst and long term sustained release of IBU are tightly controlled by concentration and volume of PCL coat in the flanking layers as reported earlier^26^. Numerous studies have reported on the efficient loading of IBU into nanoparticles, nanofibers and scaffolds yielding short term drug release for an array of biomedical applications^28,29,32,51–54^. However, very few reports are available on long term release of IBU. Recent study reported the long term sustained release of IBU from PLGA nanofibers wherein in vitro release in phosphate buffered saline showed an initial burst release of 20% followed by a lag phase with minimal drug release for 10 days. Further, the study reported a linear release phase after day 10 with a cumulative release of ~ 75–80% of IBU at the end of day 63^54^. Moreover, Immich et al.^26^ described sandwich model of PLA/ IBU, wherein IBU powder was directly layered in between PLA nanofibrous mats. Nevertheless, this model showed high initial burst release of IBU and considerably short term release kinetics.

Furthermore, all the sandwich groups along with 12% PCL-IBU fitted linearly with Korsemeyer peppas model and followed Fickians diffusion mechanism. Besides, R^2^ values observed in Higuchi diffusion model revealed the importance of polymer concentration and volume of PCL coating in the flanking layers of sandwich mat especially 12% PCLSW-2. Further, it confirms the efficient barricading to solvent penetration into the flanking PCL layers of sandwich mats. Hence 12% PCLSW-2 seems to modulate initial burst and long term sustained release by virtue of its higher concentration and volume of PCL in flanking layers amongst other groups under study. In vitro cytotoxic study revealed that all groups in the study were not cytotoxic, and the cells grew as layers on the mat for 5 days as shown by SEM images. Notably, the reported tri-layered mats with highly sustained drug release profile are easy and straight forward to fabricate when compared to other methods such as coaxial / triaxial / microfluidic methods^3,17,19–21,55–58^, hence can serve as a potential drug delivery vehicle in clinical applications. In summary, 12% PCLSW-2 with controlled initial burst and sustained release for 62 days, compared to all other groups in evaluation, seems to be best suited for delivery of IBU. In future, 12%PCLSW-2 need to be tested for its in vivo efficacy in drug delivery.

## Summary

Sandwich models of IBU loaded PCL fibrous mats were fabricated by electrospinning and extensively evaluated for their efficacy in controlled drug release. It was evident that, 12% PCL SW-1&2 showed minimal initial burst and controlled drug release for 62 days than 10% PCLSW-1&2 and 12% PCL-IBU. Further, XRD results confirmed that lipophilic drug IBU was amorphized during fabrication process which eventually enhanced the dissolution properties. Moreover, the crystallinity (%) and hydrophobicity of sandwich mats imparted by the flanking layers of PCL modulated IBU release. The outcomes of the study suggest that the volume of PCL coat and its concentration in the flanking layers efficiently modulates initial burst and long term release of IBU. Further, these mats were evaluated for cytotoxicity with C3H10T1/2 cell lines and were found to be non-cytotoxic. From the results obtained, we reinstate our hypothesis that sandwiching IBU loaded electrospun layer between hydrophobic PCL layers effectively modulates initial burst release and long term release. To conclude, 12% PCLSW-1&2 sandwich mats with comparatively desired drug release profile amongst other groups in the study need to be tested in vivo to study its potential in peripheral nerve damage and other applications.

## Experimental

### Materials

Polycaprolactone (PCL, M.W. 80000) and Ibuprofen (IBU) with 99.9% purity were purchased from Sigma Aldrich, India. Dichloromethane (DCM) (AR Grade) and dimethyl formamide (DMF) (AR Grade) were obtained from SRL Chemicals, India. Dulbeccos minimum essential media, High glucose (DMEM-HG), Dulbeccos phosphate buffered saline (DPBS, pH 7.4), fetal bovine serum (FBS), antibiotic- antimycotic solution (100X), trypsin (0.25%) EDTA (ethylenediaminetetraaceticacid) (0.02%) solution, 3-(4,5-dimethylthiazol-2-yl)-2,5-diphenyltetrazolium bromide (MTT), dimethyl sulfoxide (DMSO) were procured from Hi-Media Laboratories, India. All other chemicals were purchased from SRL Chemicals, India.

### Fabrication of fibers

**Figure 10:**
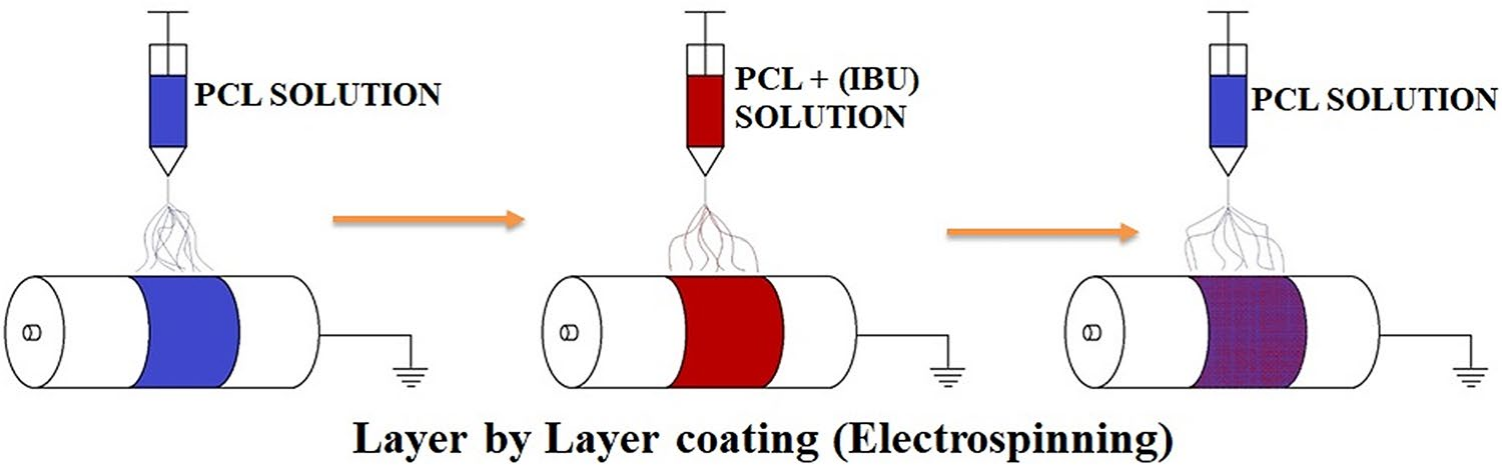
Fabrication of sandwich mats. Schematic representation of sandwich mat fabrication by sequential electrospinning.

IBU loaded PCL mat (12% PCL-IBU) was fabricated by adding IBU (4 mg/ml) in 12% (w/v) PCL solution dissolved in 1:1 ratio of DCM and DMF. Further, for sandwich mats 12% (w/v) and 10% (w/v) of PCL were dissolved in 1:1 ratio of DCM and DMF mixture. Schematic representation of layer by layer fabrication process is shown in Fig. 10. IBU loaded PCL sandwich mats were fabricated by spinning in sequence of bottom layer followed by IBU loaded middle layer and then top layer with different concentrations and volume of PCL as mentioned in Table 1. The jet orientation was adjusted manually in such a way that the fibers getting coated overlapped on each layer from bottom to top, as shown in Fig. 10. Electrospinning was carried out on ESPIN NANO (Physics instruments company, Chennai) under standard settings such as 1 ml/hour flow rate, drum to needle distance of 16 cm, 15 kV voltage, 1300 rpm drum speed, drum diameter of 70 mm, and temperature 25 °C.

## Material characterization

### Morphology and physicochemical analysis

FESEM (FEI Quanta FEG 200, USA) was employed to investigate the fiber morphology (fiber diameter, density and alignment) under high vacuum, with an accelerating voltage of 10 kV and magnification of 6000x. X-Ray Diffraction (XRD) studies were performed using Aries Research Bench Top XRD systems, Panalytical, with 2θ scans carried out from 5° to 100° and step size of 0.0430° using CuKα radiation (λ = 1.54059 Å at 45 kV and 15 mA). The full width at half-maximum height (FWHM) of X-ray diffraction peaks and their corresponding crystallite sizes were obtained using High-score plus software. Total area of all peaks and area under crystalline peaks from the spectra were calculated using Origin PRO software. Further, crystallinity (%) was calculated using the formula,

$$ Crystallinity\;percentage\;\left( {CR\% } \right) = A_{CR} /\left( {A_{CR} + A_{AM} } \right) \times 100 $$

where A_CR_—Area of Crystalline peaks, A_AM_—Area of Amorphous Peak.

Room temperature Raman spectra were acquired by using a Micro-Raman spectrophotometer, HORIBA France, LABRAM HR Evolution equipped with a solid state laser that has a wavelength of 532 nm and wavenumber ranging from 800–2000 cm^−1^. Fourier Transform Infrared (FTIR) spectra of fibrous samples were recorded in the range of 4000 to 500 cm^−1^ using Attenuated total reflection (ATR) diamond accessory of IR tracer 100 AH, Shimadzu, Japan. In addition, to investigate the precise nature of bonding of PCL with IBU in fabricated fibrous mats, Solution state ^1^H NMR spectra of all groups of sandwich mats were recorded by Bruker 500 MHz standard bore (SB) NMR spectrometer equipped with BBO probe head. Herein, Tetramethyl silane (TMS) used as internal reference.

### Contact angle measurement

For wettability analysis, Contact angle (CA) measurements were performed in a static mode with distilled water using Contact angle device (HOLMARC Opto-Mechatronics Pvt.Ltd., India). Approximately 5 µl of distilled water drop was added using micro syringe onto the top of fibrous mats and the image was acquired with high speed framing camera after 5 secs. Different areas (n = 5) on the mats were chosen and average contact angles were calculated.

### Drug release and kinetics analysis

To find the amount of drug entrapped per square centimeter, fibrous mats (1 cm × 1 cm) were dissolved in DCM and absorbance was read at the absorption maxima of 222 nm for IBU by using UV-spectrophotometer (Hitachi). The mats (1 cm × 1 cm) were sampled randomly (n = 3) from different positions on the fibrous mat to negate the variation imparted by slightly uneven coating of fibers during electrospinning. Subsequently, the concentration was derived from standard graph of IBU dissolved in DCM. Further, IBU release studies were carried out for 62 days by suspending 1 cm × 1 cm dimension of fibrous mat in 2 ml of Dulbeccos phosphate buffered saline (DPBS, pH 7.4) in triplicates (n = 3). At specific time intervals, 1 ml of sample was drawn and replaced with 1 ml of fresh DPBS; the sample was read at 222 nm. The concentration of drug released was obtained from standard graph of IBU, from which the cumulative drug release was calculated. The cumulative (%) releases were further fitted into kinetic models (Supplementary Table S3), to evaluate the mechanism of the drug release of fibrous mat.

### In-vitro cytotoxicity and cell attachment studies

The C3H10T1/2 (Mice mesenchymal stem cell line) cells lines were purchased from NCCS, Pune, India. To study the cytotoxicity of the mats, MTT assay was performed by incubating the mats with C3H10T1/2 cells for 60 h. 10^5^ cells were added to each well (12 well plates) and allowed to adhere for 12 h in growth media (DMEM-HG supplemented with 10% FBS and 1% antibiotic- antimycotic). The mats were cut into 1cm^2^ dimension and sterilized in graded series of ethanol followed by UV exposure for 30mins. Furthermore, they were suspended into their respective wells (n = 3), and the culture was continued for 60 h. After 60 h, 200 µl of MTT (4 mg/ml) dissolved in PBS was added to each well and incubated for 4 h in dark, under standard culture conditions. Subsequently, purple formazan crystals formed was solubilized in 200 µl DMSO and read using ELISA Reader (BIORAD) at 550 nm.The percentage viability was calculated using formula

$$ Percentage\;Cell\;Viability\;\left( {CV \, \% } \right) = ABS_{Test} /ABS_{Neg} \times 100 $$

where, ABS_Test_—average absorbance of treated samples, ABS_Neg_—Average absorbance of negative control.

Herewith, positive control was 12% PCL-IBU and negative control was PCL mats without drug.

Similarly, for cell attachment and proliferation studies, 10^5^ cells were added onto sterilized mats and cultured in growth medium under standard conditions for 5 days. Further, the cultured mats were fixed with 10% glutaraldehyde solution for 15mins and dehydrated with ethanol for scanning electron microscopic analysis.

## Supplementary information

Supplementary Information.
