# Supplementary material for: Fabrication of tri-layered electrospun polycaprolactone mats with improved sustained drug release profile
Source: Sci Rep. 2020 Oct 23;10:18179. doi: 10.1038/s41598-020-74885-1 (PMC7584580; doi:10.1038/s41598-020-74885-1)
Supplement: Supplementary file 1 — Supplementary Information. [file 41598_2020_74885_MOESM1_ESM.pdf]

Fabrication of tri-layered electrospun polycaprolactone mats with improved sustained drug release profile

Manjunath Kamath S.[1]*, Sridhar K.[5], Jaison D.[4], Gopinath V.[2], Mohamed Ibrahim B. K.[5], Nilkantha Gupta[1], Sundaram A.[3], Sivaperumal P.[6], Padmapriya S.[7], Shantanu Patil S.[1]

[1]Department of Translational Medicine and Research, SRM Medical College, SRMIST, Kattankulathur-603203, Tamilnadu, India

[2] Department of Medical Microbiology, Faculty of Medicine, University of Malaya, Kuala Lumpur – 50603, Malaysia

[3] Department of Pathology, SRM Medical College, SRMIST, Kattankulathur-603203, Tamilnadu, India

[4] Nanotechnology Research Center (NRC), SRMIST, Kattankulathur- 603203, Tamilnadu, India

[5] Institute of Craniofacial, Aesthetic & Plastic Surgery (ICAPS), SRM Institute for Medical Sciences (SIMS), Chennai- 600026, Tamilnadu, India

[6]Department of Pharmacology, Saveetha Dental College (SDC), Saveetha Institute of Medical and Technical Sciences, Chennai, Tamil Nadu, India

[7]Electrochemical Systems Laboratory, SRM Research Institute, SRMIST, Kattankulathur-603203, Tamilnadu, India

Supplementary Figures

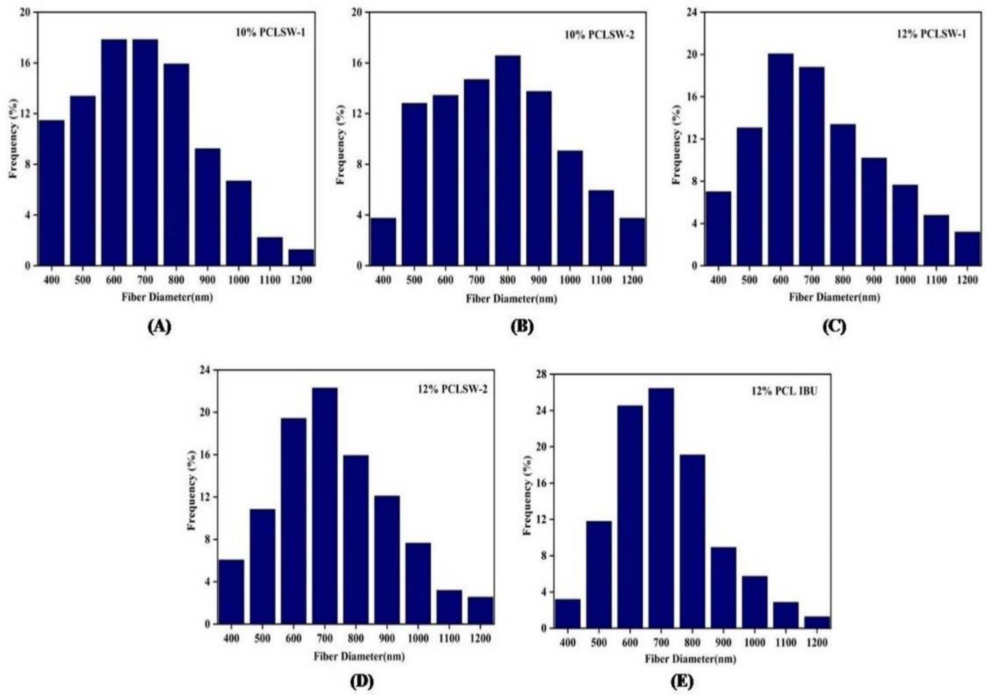

Supplementary Figure (SF 1.) Frequency distribution of fiber diameters

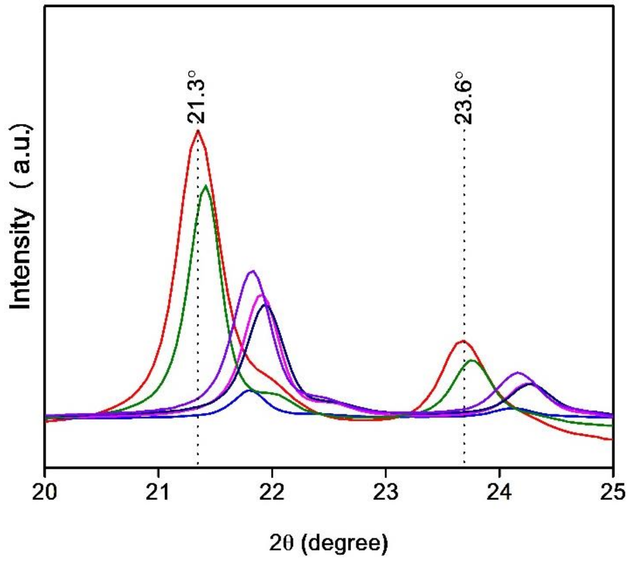

Supplementary Figure (SF 2.) Magnified image of X Ray diffraction patterns of various groups showing minor shifts.

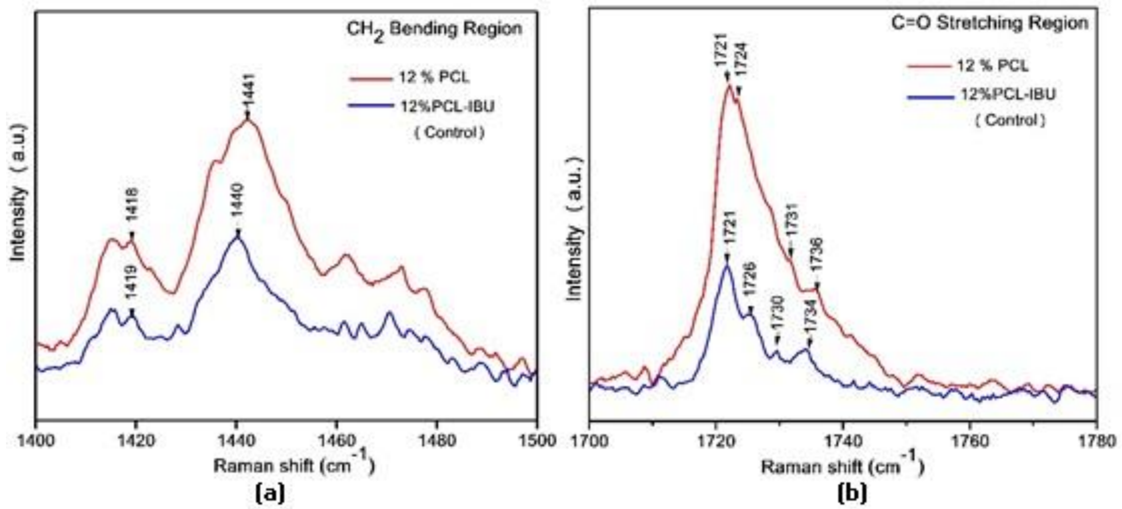

Supplementary Figure (SF 3.) Magnified Raman spectra of 12% PCL mat (without drug) and 12% PCL-IBU in the CH_2 bending and C=O stretching regions respectively.

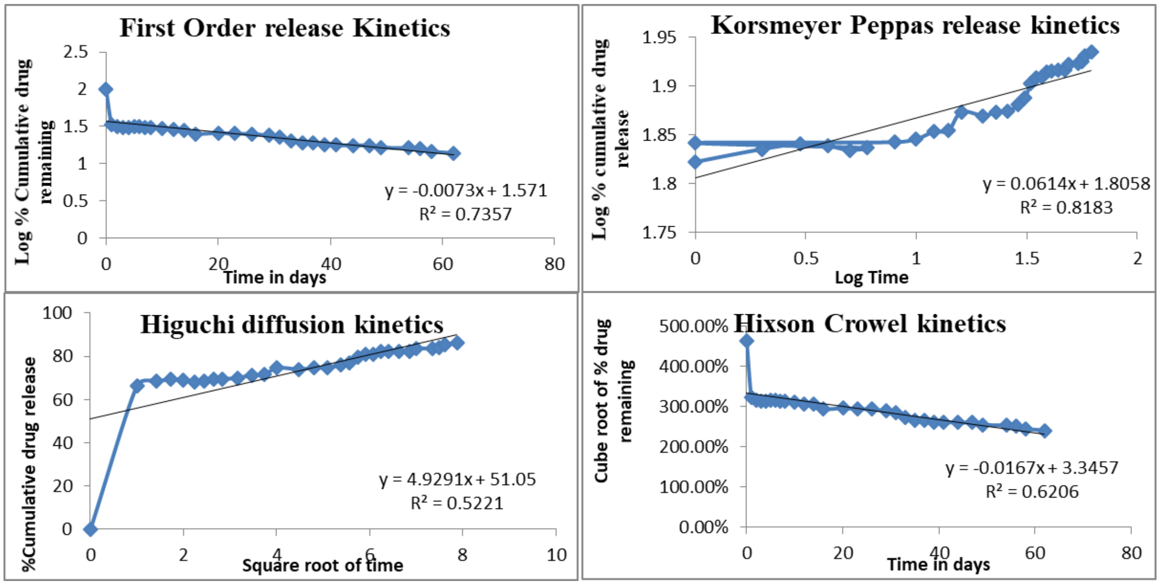

Supplementary Figure (SF 4.) 12% PCL-IBU mats release kinetics plots

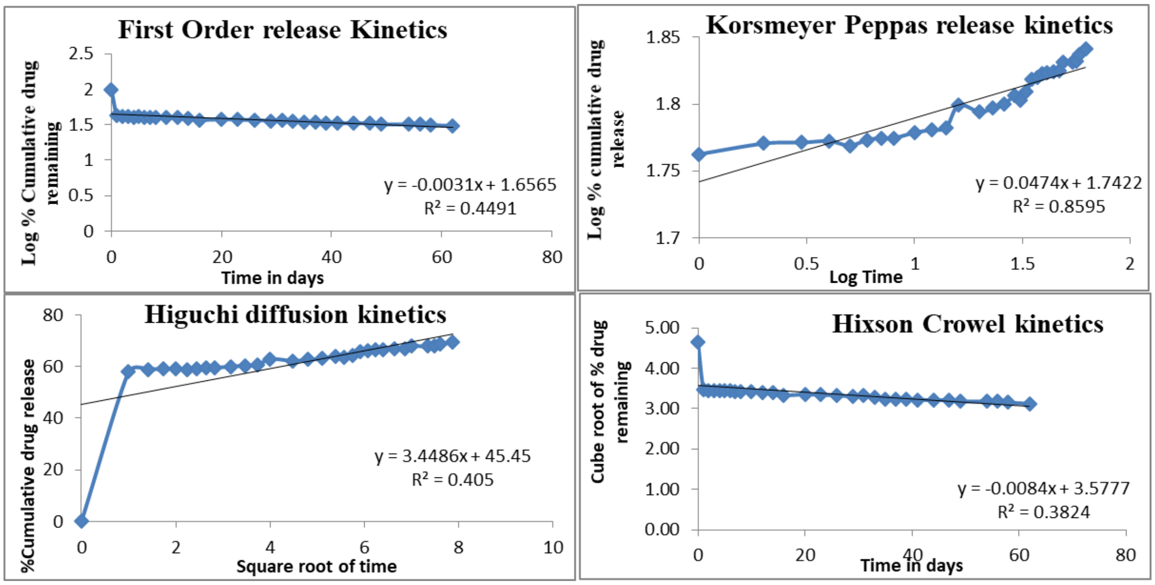

Supplementary Figure (SF 5.) 10% PCLSW-1 mats release kinetics plots

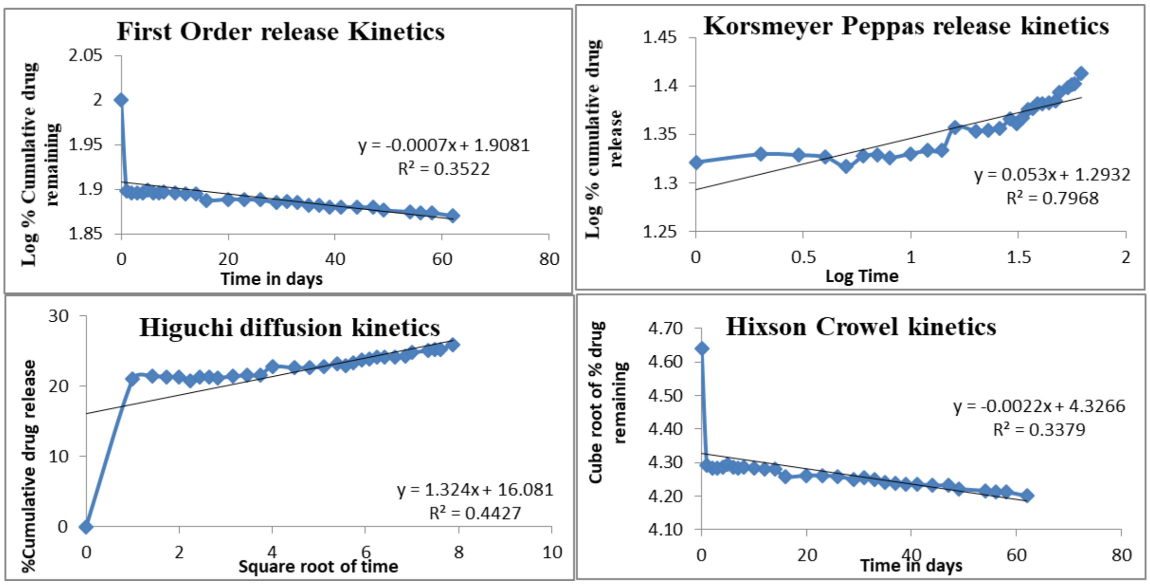

Supplementary Figure (SF 6.) 10% PCLSW-2 mats release kinetics plots

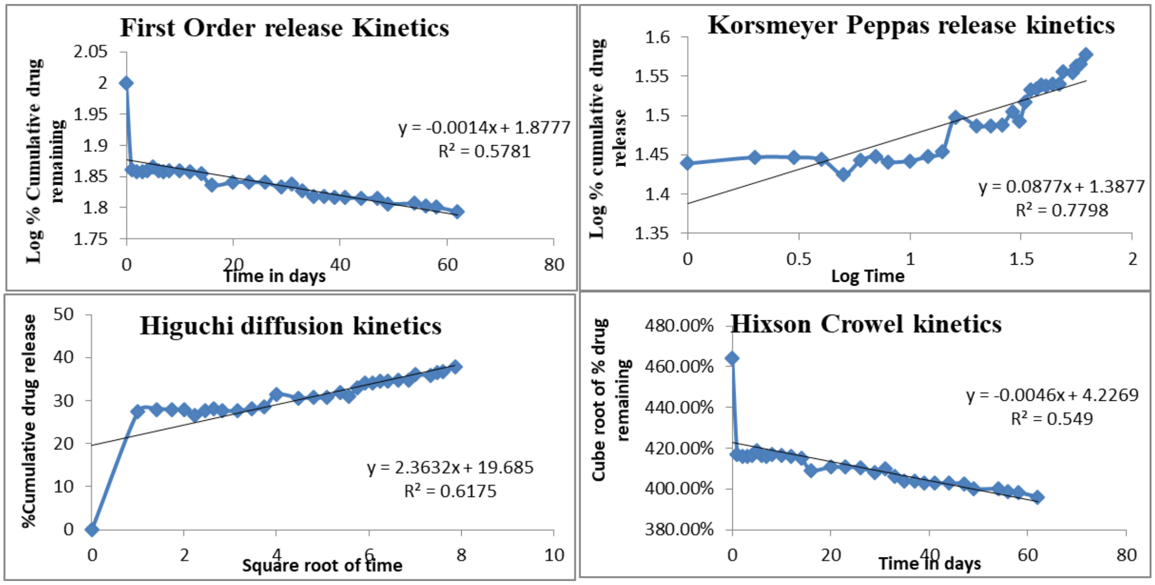

Supplementary Figure (SF 7.) 12% PCLSW-1 mats release kinetics plots

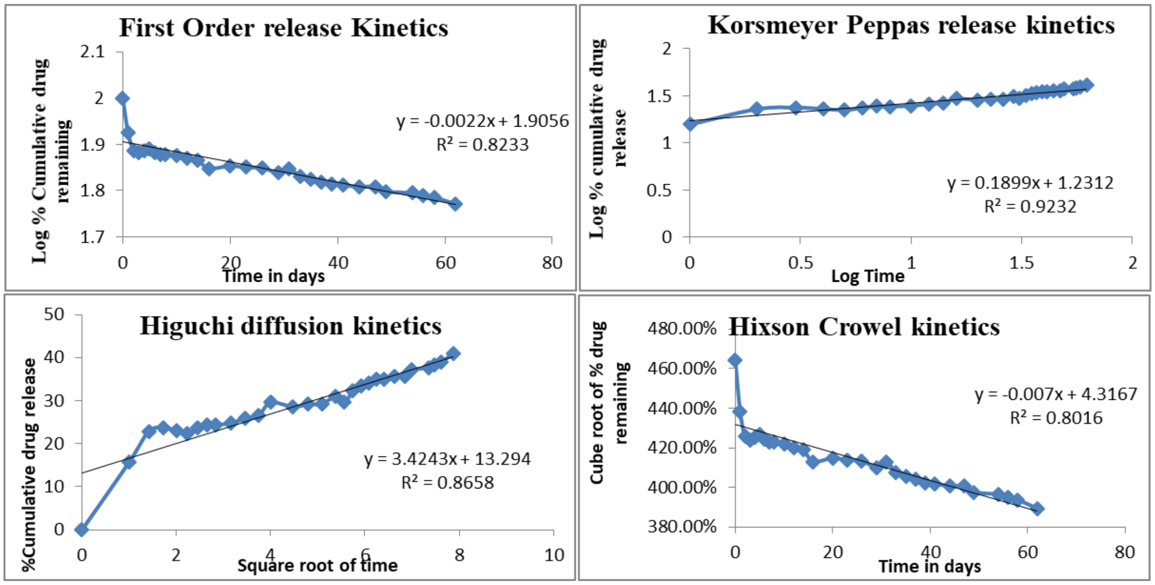

Supplementary Figure (SF 8.) 12% PCLSW-2 mats release kinetics plots

Supplementary Tables

Supplementary Table 1. Crystallinity % and crystallite sizes of all groups

Groups	Crystallite size (nm)	Crystallinity (%)
**12% PCL- IBU**	25.4	51.2
**10% PCLSW- 1**	24.4	64.6
**10% PCLSW- 2**	26.3	70.1
**12% PCLSW- 1**	22.7	63.9
**12% PCLSW- 2**	22.2	61.8

(PCL- Polycaprolactone, PCL-SW- Polycaprolactone sandwich mat, IBU-Ibuprofen)

Supplementary Table 2. Different vibrational modes of Polycaprolactone (PCL)

**Wavenumber range (cm^{-1})**	**Vibrational Mode**	**Intensity**	**Comments**
**912**<br>**956**	C-COO stretch	strong<br>weak	Crystalline
**1037**	C-C stretch	weak	Crystalline
**1064**		strong	
**1283**	CH_2 twist	strong	Crystalline
**1304**		weak	
**1418**	Methylene CH_2	weak	Crystalline
**1442**	bend	strong	
**1721**	Carbonyl C=O	Strong	Crystalline
	stretch		
**1731**	Carbonyl, C=O	weak	Amorphous
**&**	stretching,		
**1736**			

Supplementary Table 3. Mathematical drug release models with equations

Mathematical drug release models	Equations
First order release kinetics	Log Q_t = Log Q_0 + Kt/2.303<br>K – first order release constant<br>T – time in hours
Hixson Crowell Release	$3\sqrt{Q_0}$ - $3\sqrt{Qt}$ = KHC.t<br>KHC – Hixson crowell release constant
Higuchi diffusion model	$Mt/M_0 = Kt_{1/2}$<br>Mt – Amount of drug released at time t<br>M_o – Total amount of drug<br>K is Higuchi dissolution constant
Korsmeyer-peppas equation	$F=(Mt/M_0)=K_m t^n$<br>Fraction of drug released at time t<br>Mt – Amount of drug released at time t<br>M – Total amount of drug in dosage form<br>K_m – kinetic constant<br>n-Diffusion or release exponent<br>t – time in hours
